# Inter-individual variation in objective measure of reactogenicity following COVID-19 vaccination via smartwatches and fitness bands

**DOI:** 10.1038/s41746-022-00591-z

**Published:** 2022-04-19

**Authors:** Giorgio Quer, Matteo Gadaleta, Jennifer M. Radin, Kristian G. Andersen, Katie Baca-Motes, Edward Ramos, Eric J. Topol, Steven R. Steinhubl

**Affiliations:** 1 Scripps Research Translational Institute, 3344N Torrey Pines Ct Plaza Level, La Jolla, CA 92037 USA; 2 CareEvolution, 625N Main Street, Ann Arbor, MI 48104 USA

**Keywords:** Biomarkers, Infectious diseases

## Abstract

The ability to identify who does or does not experience the intended immune response following vaccination could be of great value in not only managing the global trajectory of COVID-19 but also helping guide future vaccine development. Vaccine reactogenicity can potentially lead to detectable physiologic changes, thus we postulated that we could detect an individual’s initial physiologic response to a vaccine by tracking changes relative to their pre-vaccine baseline using consumer wearable devices. We explored this possibility using a smartphone app-based research platform that enabled volunteers (39,701 individuals) to share their smartwatch data, as well as self-report, when appropriate, any symptoms, COVID-19 test results, and vaccination information. Of 7728 individuals who reported at least one vaccination dose, 7298 received an mRNA vaccine, and 5674 provided adequate data from the peri-vaccine period for analysis. We found that in most individuals, resting heart rate (RHR) increased with respect to their individual baseline after vaccination, peaked on day 2, and returned to normal by day 6. This increase in RHR was greater than one standard deviation above individuals’ normal daily pattern in 47% of participants after their second vaccine dose. Consistent with other reports of subjective reactogenicity following vaccination, we measured a significantly stronger effect after the second dose relative to the first, except those who previously tested positive to COVID-19, and a more pronounced increase for individuals who received the Moderna vaccine. Females, after the first dose only, and those aged <40 years, also experienced a greater objective response after adjusting for possible confounding factors. These early findings show that it is possible to detect subtle, but important changes from an individual’s normal as objective evidence of reactogenicity, which, with further work, could prove useful as a surrogate for vaccine-induced immune response.

## Introduction

Owing to an unprecedented effort in response to the COVID-19 pandemic, three vaccines are currently authorized and distributed in the United States: two two-dose mRNA vaccines, developed by Pfizer-BioNTech and Moderna, and one single-dose adenovirus-based vaccine, developed by Janssen/Johnson & Johnson^1–3^. The population-wide efficacy of these initial vaccine regimens, and now mRNA boosters for all, has been well established both through large-scale Phase 3 clinical trials, and reinforced by real-world data^4–8^. Although it is known that there is substantial variability in individuals’ immune response to vaccines^9^, and breakthrough infections are not uncommon after all vaccinations, including against COVID-19^10,11^, there is currently no routinely available, non-invasive method to objectively identify a specific person’s response to a vaccine beyond self-reported side effects. The Centers for Disease Control and Prevention’s (CDC) V-safe program found a majority (69%) of the 1.9 million enrolled individuals reported some systemic side effects after the second dose of a mRNA vaccine^12^, similar to the rate of early systemic adverse events reported after the second dose of both available mRNA vaccines in Phase 3 trials^4,6^. Many of the reported symptoms were consistent with systemic inflammation, including fatigue, myalgias, chills, fever and joint pain being report in the range of 25.6% to 53.9% of individuals the day following their 2nd dose^13^. The rate of systemic symptoms following a booster dose are slightly less, in general, than after a second dose, but greater than after a first dose^14^. The relationship between reactogenicity symptoms after vaccination and immune response is controversial^15^, although one study of a COVID-19 vaccine identified a direct correlation between the duration of time between a first and second vaccine dose, reactogenicity and eventual humoral immune response^16^. In addition, a recent study found a significant relationship between individual changes in physiologic parameters measured using a smart ring and ~30 day antibody levels^17^.

In this analysis from the observational, direct-to-participant, Digital Engagement and Tracking for Early Control and Treatment (DETECT) study^18,19^, we collected daily wearable sensor data from the two-weeks before and after each vaccination dose from 7298 volunteers who reported receiving at least one dose of the vaccine (6803 received both doses of a mRNA vaccine). We hypothesized that there are digital, objective biomarkers of reactogenicity that could be identified via the detection of subtle deviations from an individual’s normal resting heart rate (RHR). We also explored individual behavioral changes following vaccination via measured changes in a person’s routine sleep and activity. Through exploring individual and vaccine characteristics that might influence reactogenicity, we identified significant associations between individual changes in RHR and prior COVID-19 infection status or vaccine type (Moderna versus Pfizer/BioNTech), which have been previously reported to correlate with subjective symptoms^13,20^.

## Results

### Changes in resting heart rate

At least one mRNA vaccination to date was reported by 7,298 participants in the DETECT study. After applying the exclusion criteria discussed in Methods, we included a total of 5674 (78%) individuals for the analysis of changes in their RHR. Of them, 314 (5.5%) reported having been previously diagnosed with COVID-19 infection, 2388 (42%) received the Moderna vaccine and 3286 (58%) received the Pfizer-BioNTech vaccine. In all, 4628 (63%) and 5691 (78%) participants contributed adequate data—as discussed in Methods—to evaluate changes in activity and sleep, respectively (Supplementary Table 1).

**Fig. 1:**
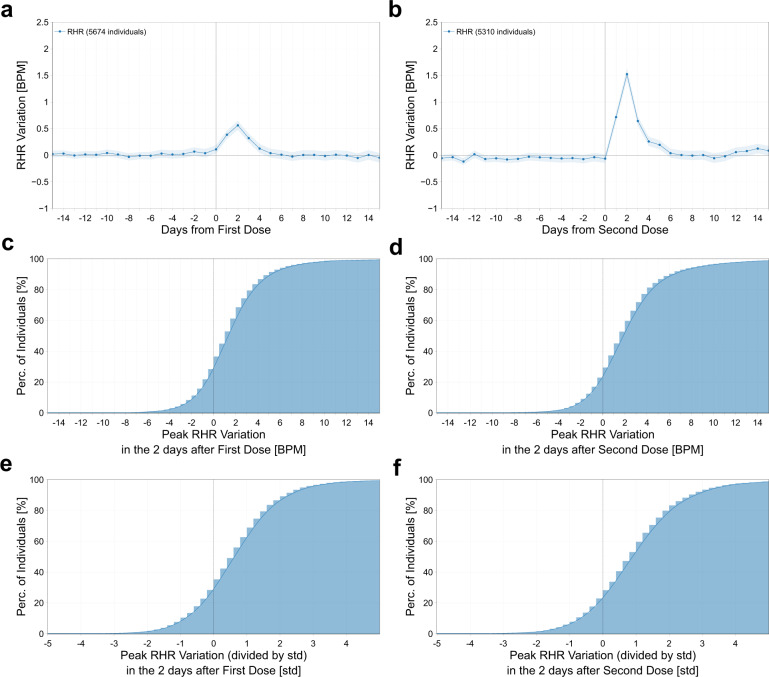
Peak resting heart rate changes post vaccination. Mean and 95% confidence interval of the absolute individual changes in resting heart rate (in BPM) with respect to the individual baseline around the date of vaccination (day 0), for the first dose of the vaccine (**a**) and for the second dose (**b**). The cumulative distribution of the maximal variation in resting heart rate in the 2 days after the first (**c**) and second (**d**) vaccine dose, and the variation normalized by the individual standard deviation (**e**, **f**).

We observed that the average RHR significantly increased the day following vaccination, reaching a peak on day 2 with a population mean increase of +0.56 (CI: [0.48, 0.65], one-sided *t*-test, *p* < 0.001) and +1.52 (CI: [1.42, 1.63], *p* < 0.001) beats per minute (BPM) with respect to baseline, following the first and second dose, respectively. The average RHR did not return to baseline until day 4 after the first dose and day 6 after the second (Fig. 1a, b). The majority of vaccinated individuals, 71% and 76% after first and second dose, respectively, experienced an increase from their normal RHR in the two days following the vaccine (Fig. 1c, d). This increase in RHR was greater than one standard deviation above individuals’ normal daily pattern in 37% of participants after their first vaccine dose, and 47% after their second (Fig. 1e, f).

**Fig. 2:**
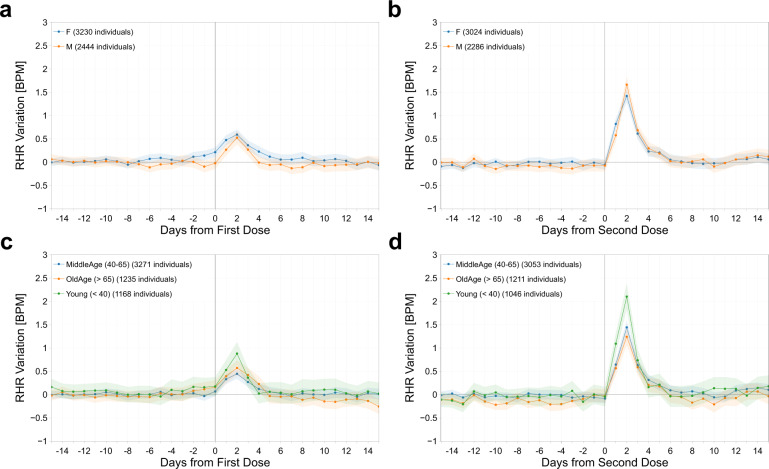
Resting heart rate changes by sex and age. Mean and 95% confidence interval of the absolute individual changes in resting heart rate (in BPM) with respect to the individual baseline around the date of vaccination (day 0), for the first dose of the vaccine (**a**), (**c**), and for the second dose (**b**), (**d**), for all individuals grouped by gender (**a**), (**b**) and age (**c**), (**d**).

We explored several participant and vaccine characteristics that could impact immune response (Table 1). Women experienced higher RHR changes with respect to baseline in the 5 days following vaccination after the first dose only (two-sided *t*-test, *p* = 0.014 and *p* = 0.646 for first and second doses, respectively). In contrast, we found that RHR responses vary by age, with individuals age <40 years having the greatest increase in RHR (Fig. 2). Age <40 years was associated with a significantly higher RHR increase than 40+ years, but only after the second dose of the vaccine (average 0.79 BPM, *p* = 0.011).

**Table 1:** Mean changes in RHR, sleep and activity metrics with respect to the individual baseline in the day of vaccination and the following 4 days, for first and second dose.

	After First Dose				After Second Dose			
	Mean	(95% CI)	Individuals	*p*-value	Mean	(95% CI)	Individuals	p-value
**RHR Variation [BPM]**								
Overall	0.3	(0.24, 0.36)	5674		0.61	(0.55, 0.68)	5310	
Female	0.37	(0.28, 0.45)	3230	0.014	0.6	(0.51, 0.69)	3024	0.646
Male	0.21	(0.12, 0.30)	2444		0.63	(0.53, 0.73)	2286	
Moderna	0.41	(0.31, 0.51)	2388	**0.003**	0.85	(0.75, 0.96)	2244	**< 0.001**
Pfizer/BioNTech	0.22	(0.14, 0.30)	3286		0.44	(0.35, 0.53)	3066	
Young (< 40)	0.39	(0.22, 0.55)	1168	0.172	0.79	(0.61, 0.97)	1046	0.011
MiddleAge (40−65)	0.25	(0.17, 0.33)	3271	0.049	0.59	(0.50, 0.68)	3053	0.465
OldAge (> 65)	0.37	(0.25, 0.48)	1235	0.308	0.52	(0.40, 0.63)	1211	0.119
Prev. Positive	0.66	(0.32, 0.98)	314	**0.008**	0.43	(0.14, 0.73)	325	0.181
No prior infect.	0.28	(0.22, 0.34)	5360		0.62	(0.56, 0.70)	4985	
**Sleep Variation [min]**								
Overall	2.98	(1.88, 4.07)	4628		6.36	(5.17, 7.52)	4341	
Female	3.23	(1.72, 4.76)	2647	0.608	6.59	(4.93, 8.26)	2485	0.648
Male	2.66	(1.14, 4.24)	1981		6.02	(4.37, 7.68)	1856	
Moderna	3.54	(1.94, 5.12)	1941	0.399	8.68	(6.81, 10.60)	1827	**0.001**
Pfizer/BioNTech	2.57	(1.15, 4.06)	2687		4.67	(3.18, 6.22)	2514	
Young (< 40)	2.42	(−0.06, 4.98)	980	0.619	8.98	(6.14, 11.73)	870	0.033
MiddleAge (40−65)	3.38	(1.93, 4.78)	2673	0.406	5.95	(4.39, 7.51)	2508	0.418
OldAge (> 65)	2.46	(0.20, 4.70)	975	0.612	5.13	(2.74, 7.54)	963	0.274
Prev. Positive	5.67	(0.30, 11.26)	267	0.219	3.08	(−1.96, 8.39)	275	0.164
No prior infect.	2.81	(1.73, 3.90)	4361		6.57	(5.37, 7.83)	4066	
**Steps Variation**								
Overall	71.05	(21.28, 122.88)	5691		−345.46	(−395.90, −296.70)	5324	
Female	76.31	(11.02, 144.80)	3240	0.812	−408.01	(−472.94, −342.64)	3026	**0.005**
Male	63.64	(−12.14, 136.55)	2451		−263.1	(−342.31, −187.08)	2298	
Moderna	4.96	(-69.64, 78.74)	2394	0.027	−540.34	(−619.86, −462.33)	2247	**< 0.001**
Pfizer/BioNTech	119.28	(51.58, 186.82)	3297		−202.93	(−266.28, −139.62)	3077	
Young (< 40)	155.84	(48.16, 265.02)	1167	0.088	−393.19	(−506.19, −281.53)	1039	0.360
MiddleAge (40−65)	110.72	(42.10, 180.96)	3289	0.069	−330.98	(−397.58, −262.03)	3078	0.493
OldAge (> 65)	−114.07	(−203.18, -25.68)	1235	**< 0.001**	−342.07	(−438.98, −246.45)	1207	0.953
Prev. Positive	−60.63	(−248.50, 132.07)	316	0.212	−320.72	(−510.56, −127.00)	329	0.803
No prior infect.	78.57	(27.61, 131.51)	5375		−347.21	(−398.12, −296.45)	4995	

Individuals are divided into groups based on gender, age, COVID-19 vaccine type and previously reported COVID-19-positive test. Statistically significant *p*-values after Holm-Bonferroni correction have been highlighted in bold.

**Fig. 3:**
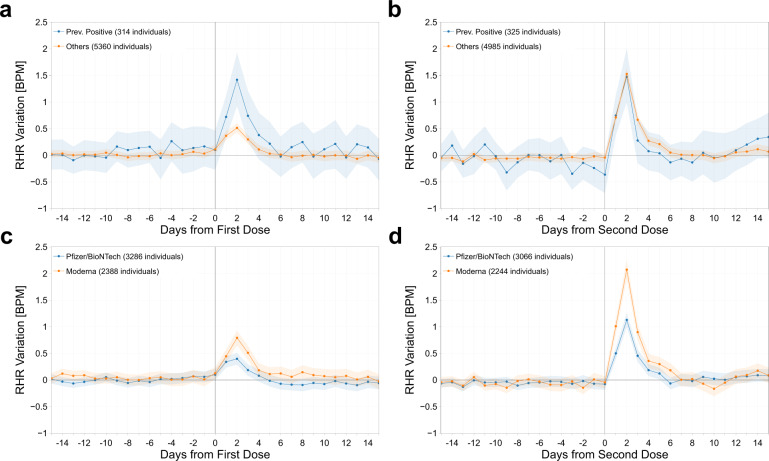
Resting heart rate changes by previous COVID-19 infection and vaccine received. Mean and 95% confidence interval of the absolute individual changes in resting heart rate (in BPM) with respect to the individual baseline around the date of vaccination (day 0), for the first dose of the vaccine (**a**), (**c**), and for the second dose (**b**), (**d**), based on prior COVID-19 infection (**a**), (**b**), and based on the type of mRNA vaccine received, either Pfizer-BioNTech or Moderna vaccines (**c**), (**d**).

Prior COVID-19 infection was associated with a significantly higher RHR increase after the first vaccine dose relative to those without prior infection (average 0.66 versus 0.28 BPM, *p* = 0.008), with no difference after the second dose (0.43 versus 0.62, *p* = 0.181) (Fig. 3a, b) (Table 1). The changes in RHR for individuals who received the Moderna vaccine were significantly greater than those who received the Pfizer-BioNTech vaccine, after both the first (0.41 versus 0.22, *p* = 0.003) and second doses (0.85 versus 0.44, *p* < 0.001) (Fig. 3c, d) (Table 1). Since multiple hypothesis have been tested, we applied the Holm–Bonferroni method for family-wise error rate corrections, which is highly conservative. All the results discussed above remained significant, except for RHR variation of women after the first dose.

Although a direct comparison is not possible, as the Johnson & Johnson vaccine was reported by only 437 individuals (with 326, 262 and 326 individuals with sufficient RHR, sleep and activity data, respectively) in our cohort, changes comparable to the ones observed after the second dose of the two mRNA vaccines were detected, which is consistent with previous reports of subjective reactogenicity experienced after the single dose of the Johnson & Johnson vaccine^21^ (Supplementary Fig. 1).

A multiple regression model was used to adjust for potential confounding factors. Prior COVID-19 infection was independently associated with a higher RHR increase after the first dose, with estimated marginal mean of 0.68 (CI: [0.41, 0.95]) versus 0.30 (CI: [0.22, 0.37]) BPM, *p* = 0.007; and no significant difference after the second dose, 0.53 (CI: [0.25, 0.81]) versus 0.72 (CI: [0.64, 0.80]), *p* = 0.188, after adjusting for age, gender, device, and vaccine type. Similarly, the Moderna vaccine was also independently associated with a higher RHR increase after both doses (with respect to Pfizer-BioNTech), with estimated marginal mean of 0.58 (CI: [0.42, 0.75]) versus 0.39 (CI: [0.24, 0.55]), *p* = 0.003 for first dose, and 0.84 (CI: [0.67, 1.01]) versus 0.42 (CI: [0.26, 0.58]), *p* < 0.001 for second dose, after adjusting for age, gender, device, and prior COVID-19 infection. Female sex was independently associated with a higher RHR increase after the first dose, with estimated marginal mean of 0.58 (CI: [0.42, 0.74]) versus 0.43 (CI: [0.26, 0.60]) BPM, *p* = 0.021; and no significant difference after the second dose, after adjusting for age, device, vaccine type, and prior COVID-19 infection. Young age (<40) was independently associated with a higher RHR increase after the second dose, with estimated marginal mean of 0.84 (CI: [0.65, 1.04]) versus 0.60 (CI: [0.45, 0.76]) BPM, *p* = 0.005; and no significant difference after the first dose, after adjusting for gender, device, vaccine type, and prior COVID-19 infection. Owing to the different algorithms used to estimate the RHR by different devices, we observed higher changes from Apple devices on average. This difference was significant after the second vaccine dose only (*p* < 0.001). We assessed the interaction between age and gender but did not find it to be significant (*p* = 0.698 and *p* = 0.182 for first and second dose, respectively) (Supplementary Table 2).

### Behavioral changes—daily activity and sleep

**Fig. 4:**
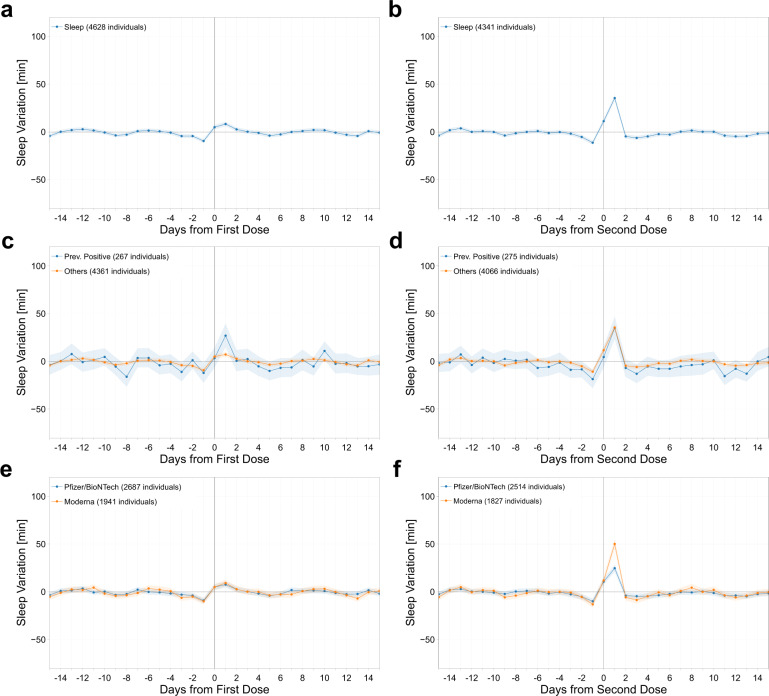
Sleep metric changes post vaccination. Mean and 95% confidence interval of the absolute individual changes in sleep metric (in minutes) with respect to the individual baseline around the date of vaccination (day 0), for the first dose of the vaccine (**a**), (**c**), (**e**), and for the second dose (**b**), (**d**), (**f**), for all individuals vaccinated (**a**), (**b**), for individuals previously tested positive to COVID-19 (**c**), (**d**), and for individuals vaccinated with the Pfizer-BioNTech or Moderna vaccines (**e**), (**f**).

**Fig. 5:**
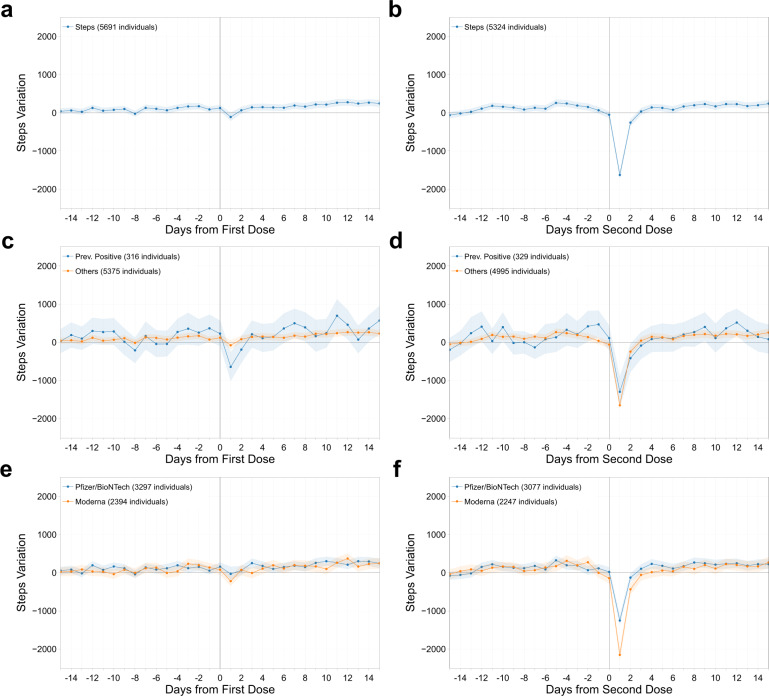
Activity metric changes post vaccination. Mean and 95% confidence interval of the absolute individual changes in activity metric (number of steps) with respect to the individual baseline around the date of vaccination (day 0), for the first dose of the vaccine (**a**), (**c**), (**e**), and for the second dose (**b**), (**d**), (**f**), for all individuals vaccinated (**a**), (**b**), for individuals previously tested positive to COVID-19 (**c**), (**d**), and for individuals vaccinated with the Pfizer-BioNTech or Moderna vaccines (**e**), (**f**).

We observed that normal activity and sleep patterns among participants were minimally affected by the first dose of the vaccine, with no decrease in number of steps and a mean increase of only 8 min (CI: [6, 11]) of sleep in the day following the vaccine. However, a significant decrease in activity (–1628 steps, CI: [–1726, –1530]) and increase in sleep (35 min, CI: [33, 39]) relative to baseline were observed on day 1 after the second vaccine dose, both of which returned to baseline by day 2 (Figs. 4 and 5). Interestingly, changes in sleep and activity were not highly correlated to changes in RHR. The Pearson correlation coefficient between the average changes in RHR and sleep was –0.05 and –0.01 after first and second dose, and between RHR and activity was 0.02 and 0.01 after first and second dose.

## Discussion

In the present study, we demonstrate that it is possible to recognize physiologic manifestations of reactogenicity to COVID-19 vaccination through individual changes in RHR. While the absolute changes are small and would be unrecognizable in a standard healthcare setting, these findings, and their consistency with reported subjective reactogenicity, highlight the value of wearable sensors to detect subtle deviations from an individual’s “normal.” With knowledge of an individual’s pre-vaccine “normal”^22–26^, we were able to identify changes in RHR of at least one standard deviation above an individual’s usual, pre-vaccine RHR pattern in ~half of individuals following their second vaccine dose. As there are currently no non-invasive, objective means of detecting a response to vaccines in a scalable manner, these findings, along with other recent work^17^, provide a potential novel mechanism to identify individuals with either a suboptimal or exaggerated immune response to a vaccine.

Individual response to vaccination is remarkably complex, incorporating components of innate, humoral and cell-based immune system. A study of response to yellow fever vaccination found significant modulation of expression in 97 genes in the days following vaccination^27^. Modern improvements in a range of analytic tools have enabled a system biological approach to better understand the immune responses to vaccination through potentially defining molecular signatures that may predict vaccine-induced immunity^28^. While a recent study identified that neutralizing levels can be predictive of immune protection^29^, there are no commercially available tests for neutralizing antibodies to the spike protein or its components S1, S2, RBD, that would provide quantitative evidence of an immune response. Beyond humoral immunity, the early T-cell spike-specific response has recently been shown to be important^30^, yet is only rarely assessed. All these tests would require at least one blood test following vaccination, which limits its implementation to primarily small-scale research programs and would certainly not be feasible to implement in hopes of identifying the minority of individuals with a suboptimal response to an approved vaccine. Accordingly, it is presently impossible to identify, at scale, the level of protection an individual acquires after vaccination^31^.

Currently available in the United States mRNA vaccines (Pfizer-BioNTech and Moderna) and adenovirus vaccine (Johnson & Johnson) elicit an inflammatory response through immune cell activation leading to the production of Type 1 interferon and the release of multiple inflammatory mediators^32^. Vaccination has been shown to stimulate the production of neutralizing antibodies, activate virus-specific CD4+ and CD8+ T cells, and lead to the robust release of immune-modulatory cytokines in the days that follow a first, and especially a second dose of the mRNA vaccines^33^. Beyond rapid stimulation of innate immunity via adjuvant stimulation, prior studies of mRNA vaccines have shown peak production of the vaccine-induced antigen protein to occur as soon as 6 h after vaccination, suggesting that an inflammatory response begins within hours of vaccination^34^.

Heart rate increases in the setting of systemic inflammation^35^. Consistent with that, we identified a rapid rise in heart rate the day after vaccination, and one that was more robust after the second dose, unless the participant had prior COVID-19 infection^20^, mirroring the significantly higher incidence of systemic symptoms following the second dose found in V-safe^36^. We also observed a more pronounced increase after a Moderna vaccine, in accordance to a recent analysis of V-safe data that identified a higher incidence of side effects relative to those receiving the Pfizer-BioNTech vaccine, especially after the second dose^13^. This difference in both objective and subjective measures of reactogenicity is likely related to the higher dose (100 micrograms) of the Moderna versus the Pfizer-BioNTech vaccine (30 micrograms), which may also explain the greater humoral immune response following the Moderna vaccine and its greater long-term protection from infection compared to the Pfizer-BioNTech vaccine^37–39^. Similarly, the significantly greater heart rate response at the time of vaccination, especially the first dose in those with prior infection, is consistent with a greater immune response for these individuals^40^.

Immunosenescence, or waning response to vaccination as someone ages, has been described for many vaccines and is a concern for COVID-19, although studies in nursing home residents have shown equivalent efficacy as in broader populations^41,42^. We found that individuals in the younger age group (<40 years) had a significantly higher RHR response to the second dose compared to older individuals. Overall, women showed a greater change in RHR after the first dose, and accordingly reported more side effects to V-Safe compared to men^36^. Immune response to other vaccines has varied by gender, possibly because of differences in hormones, genetics, or differences in dosing by weight. A prior flu vaccine study found that vaccine induced immunity in mice was increased by estradiol in females and decreased by testosterone in males^43^ and that as age increased, sex differences in vaccine efficacy was declined. Although the RHR differences after the second dose were not significant, it is possible that a greater change would be identified when younger age group’s vaccination data are included.

While we found that multiple observable variables (age, gender, previously COVID-19 infection, device used, vaccine type) influence observed changes in RHR as a measure of reactogenicity, these variables can explain only 1.2% of the variance in terms of average changes in RHR (and less than 21.1% of the variance in terms of peak changes in RHR). It is possible that with further investigation it may be found that interindividual variation in RHR response to vaccines may correlate with individual immune response. If so, this would suggest that wearables could offer a way to easily quantify someone’s immune response to a vaccine and allow for changes in preventative strategies, such as giving an early booster shot^44^.

The presence of a fever has previously been shown to be associated with an increase in heart rate, with an ~8.5 BPM increase for every degree Celsius increase in body temperature^45^. While ~30% of V-safe participants reported having a fever after their second dose and ~9% after the first, we showed that the vast majority of participants experienced an increase in RHR after both vaccination doses, suggesting that inflammation unassociated with an elevated temperature also influences heart rate, as has been previously shown^35^.

Beyond the changes in RHR, we also found objective changes in people’s sleep and activity levels, especially after their second vaccine dose. Although inflammation has also been shown to lead to an increase in sleep and a decrease in activity, it is difficult to distinguish the degree of change directly due to inflammation rather than the conscious act of planning to have a restful day following receiving a vaccine^46,47^. Future work will provide additional information regarding the value of tracking objective changes in sleep and activity and a measure of reactogenicity and, potentially, vaccine response.

The data collected as part of the DETECT study depends entirely on the participants’ willingness to use their wearable device and accurately reporting vaccination date and type. While we do not have direct control on self-reported information, the DETECT app provides an intuitive tool to self-report vaccination information, and an optional reminder to report information on the second dose after first one has been reported. While the information collected may not be as accurate as in a controlled laboratory setting, we rely on previous work confirming that self-reported symptoms and sensor data provide valuable information^48–50^. Only daily sensor data is considered in this analysis, excluding intra-day data provided by some wearable sensor. These once-a-day values are indeed more stable and less affected by independent confounders like the specific activity performed by the individual during the day. In addition, we did not capture whether participants self-treated with anti-inflammatory and/or anti-pyretic medications, which would likely influence the measured physiologic response. Furthermore, the population in the DETECT study that has received a COVID-19 vaccine may not be representative of the population of the United States, as the study is open to individuals who have access to a wearable device technology^51^. While research has found no racial or ethnic variation in smartwatch or activity tracker usage in the U.S., they are less commonly utilized by older individuals, those in the lowest socioeconomic tertile, and lower educational attainment^52^. It is also possible that some participants had prior COVID-19 that went undiagnosed, which may have impacted their immune response to the first dose.

Fitness bands and smartwatches are owned by approximately 1 in 5 American adults^52^. By taking advantage of these user-friendly devices we were able to recognize subtle, but significant deviations from an individual’s unique, normal resting heart rate following vaccination. We were also able to demonstrate substantial interindividual variability in that heart rate increase that was related, in part, to the mRNA vaccine type and prior COVID-19 infection in our population, both characteristics associated with subjective reactogenicity and immune response, as reported by others. With further study, not only might this individual information provide reassurance for vaccinees who do not experience any symptoms, but correlation with the humoral and cellular immune response may indicate digital tracking as a useful surrogate. Noteworthy is the potential, when further validated with immunologic assays, to identify the minority of people who do not have an adequate immune response to vaccines, and who may benefit by more in-depth assessment and re-vaccination, such as recently identified in individuals with autoimmune conditions^53^.

## Methods

### DETECT study population

DETECT is an app-based longitudinal prospective study, which has enrolled 39,701 individuals so far from the United States (from March 25, 2020 to September 12, 2021) who have donated their wearable data, self-reported symptoms when ill, viral testing results, and vaccination dates/type. The protocol for DETECT was reviewed and approved by the Scripps Office for the Protection of Research Subjects (IRB 20–7531). All participants in the study provided informed consent electronically.

Among DETECT participants, 7728 have reported receiving at least one dose of the vaccine (7298 first dose, 6803 both first and second dose, 437 single dose), 57% were female and their median age was 53 (inter quartile range, IQR 42–64).

Individuals who had been vaccinated with the single-dose Johnson & Johnson vaccine were excluded from the analysis as there were too few (437 individuals) to allow for a meaningful comparison, but their results are reported in Supplementary Fig. 1. We have included in the analysis individuals wearing a Fitbit device (76%) and an Apple watch (20%), while 152 individuals with other devices were not included in this analysis. We also excluded 75 participants who reported a vaccine date before Dec. 11, 2020—the official date of the first US vaccine intake—and 16 participants who did not report age or gender. Individuals were excluded if they had less than 4 days of recording in the 2 weeks before vaccination, or less than 3 of the 5 days after vaccination, or less than 14 days during the baseline period (from 60 days to 7 days before vaccination). A number of individuals were excluded in the calculation of RHR (1552), sleep (2598) and activity (1535) metrics because of missing data.

### Data processing and statistical analysis

For each individual, we calculated the average of the absolute changes of RHR, sleep and activity with respect to their individual baseline, which we have previously shown to be relatively stable for an individual over time, but to vary substantially between individuals^22,23^. A single daily value is considered valid only if the device was worn for more than 15 h during the day. The RHR was based on the value of heart rate that would be obtained in a supine position immediately after waking but before getting out of bed for Fitbit devices^22^, and by considering heart rate values over the day by a proprietary algorithm for Apple watches. The individual baseline was calculated using the period from 60 days to 7 days before vaccination, using a decreasing exponential (with exponent *α* = 0.05) to reduce the weight of days farthest in the past. The baseline for the RHR was calculated as

1 $${{{\mathrm{RHRbaseline}}}} = \frac{{\mathop {\sum }\nolimits_{d = - 60}^{ - 7} e^{{\upalpha d}}RHR(d)}}{{\mathop {\sum }\nolimits_{d = - 60}^{ - 7} e^{{\upalpha d}}}}$$

while the RHR metric was

2 $${{{\mathrm{RHRmetric}}}}\left( d \right) = {\mathrm{RHR}}\left( d \right) - {\mathrm{RHR}}{\mathrm{baseline}}$$

The sleep and activity metrics were calculated accordingly using the total time asleep and the number of steps recorded by the sensor in the 24 h, respectively. In the figures, the mean (over all individuals) and the 95% confidence interval for each metric (RHR, sleep and activity) in the 15 days before and after the first and second dose of the vaccine are represented. The cumulative distribution of the maximal variation in RHR in the 2 days after the vaccines is also represented.

The cohort of vaccinated individuals (with first and second vaccine doses, treated separately) was then split into subgroups according to gender, age (<40, 40–60, >60), vaccine type received, and if they previously reported a COVID-19-positive test. For each subgroup and for each metric, the mean (over all individuals) of the individual average value (calculated considering the day of vaccination and the following 4 days), with the corresponding confidence interval was calculated. The 95% confidence interval in the calculation of the mean is obtained with a bootstrap method with 10,000 iterations.

The demographic characteristics of these groups are reported in Supplementary Table 1.

Unless stated otherwise, all the reported *p*-values refer to a two-sided t-test to quantify statistical difference among different groups (Table 1), and to a chi-squared test to evaluate significant changes in the frequency of observation in each group (Supplementary Table 1). The *p*-value associated with each age subgroup has been calculated by comparing the corresponding subgroup with its complement (Table 1). In order to correct for occurrence of type I errors when performing multiple hypotheses tests, we applied family-wise error rate corrections to the results (Table 1) using the Holm–Bonferroni method, which is highly conservative if there is a large number of tests or the test statistics are positively correlated. Family-wise error rate corrections adjust *p*-values derived from multiple statistical tests among a specified group. In our analysis, three groups were identified: RHR, activity and sleep related comparisons. We have reported which test becomes not statistically significant after the Holm–Bonferroni correction.

A multiple linear regression model was used to calculate the estimated marginal means after adjusting for potential confounding variables^54^. A *t*-test was used to assess significant coefficients and the associated *p*-values (Supplementary Table 2).

### Reporting summary

Further information on research design is available in the Nature Research Reporting Summary linked to this article.

## Supplementary information

Supplementary

Reporting Summary

## Data Availability

All interested investigators will be allowed access to the analysis data set after approval of a proposal by a responsible authority at Scripps and with a data access agreement, pledging to not re-identify individuals or share the data with a third party. All data inquiries should be initially addressed to the corresponding author.

## Code availability

Further information on research design and on all software packages adopted in the analysis is available in the Life Sciences Reporting Summary linked to this article.
