# Supplementary material for: Inter-individual variation in objective measure of reactogenicity following COVID-19 vaccination via smartwatches and fitness bands
Source: NPJ Digit Med. 2022 Apr 19;5:49. doi: 10.1038/s41746-022-00591-z (PMC9019018; doi:10.1038/s41746-022-00591-z)
Supplement: Supplementary file 1 — Supplementary [file 41746_2022_591_MOESM1_ESM.pdf]

SUPPLEMENTARY TABLES AND FIGURES

		FirstDose					SecondDose				
		**Age Median [IQR]**	**Female**	**Young (< 40)**	**Prev. Positive**	**Fitbit**	**Age Median [IQR]**	**Female**	**Young (< 40)**	**Prev. Positive**	**Fitbit**
**RHR Variation [ppm]**	**Overall**	54<br>[43 - 64]	56.90%	20.60%	5.50%	75.50%	54<br>[43 - 65]	56.90%	19.70%	6.10%	75.40%
	**Moderna**	55<br>[44 - 66]	57.3%<br>p=0.621	19.4%<br>p=0.062	4.8%<br>p=0.050	75.0%<br>p=0.506	56<br>[44 - 66]	57.6%<br>p=0.414	18.5%<br>p=0.064	6.0%<br>p=0.742	75.0%<br>p=0.561
	**Pfizer/BioNTech**	53<br>[42 - 63]	56.6%<br>p=0.621	21.5%<br>p=0.062	6.1%<br>p=0.050	75.8%<br>p=0.506	53<br>[42 - 64]	56.5%<br>p=0.414	20.6%<br>p=0.064	6.2%<br>p=0.742	75.7%<br>p=0.561
	**Prev. Positive**	51<br>[40 - 60]	61.5%<br>p=0.107	25.5%<br>p=0.033	100.00%	77.4%<br>p=0.455	52<br>[41 - 61]	61.8%<br>p=0.075	23.4%<br>p=0.098	100.00%	78.8%<br>p=0.168
	**Others**	54<br>[43 - 64]	56.7%<br>p=0.107	20.3%<br>p=0.033	0.00%	75.4%<br>p=0.455	55<br>[43 - 65]	56.6%<br>p=0.075	19.5%<br>p=0.098	0.00%	75.2%<br>p=0.168
**Sleep Variation [min]**	**Overall**	54<br>[42 - 64]	57.20%	21.20%	5.80%	86.50%	54<br>[43 - 64]	57.20%	20.00%	6.30%	86.20%
	**Moderna**	55<br>[43 - 65]	58.0%<br>p=0.356	19.9%<br>p=0.086	5.1%<br>p=0.111	86.2%<br>p=0.676	56<br>[44 - 66]	58.0%<br>p=0.432	18.7%<br>p=0.069	6.1%<br>p=0.593	85.8%<br>p=0.485
	**Pfizer/BioNTech**	53<br>[42 - 63]	56.6%<br>p=0.356	22.1%<br>p=0.086	6.3%<br>p=0.111	86.7%<br>p=0.676	53<br>[42 - 64]	56.7%<br>p=0.432	21.0%<br>p=0.069	6.5%<br>p=0.593	86.6%<br>p=0.485
	**Prev. Positive**	51<br>[40 - 60]	61.0%<br>p=0.212	26.2%<br>p=0.045	100.00%	86.9%<br>p=0.926	52<br>[41 - 60]	61.8%<br>p=0.128	23.3%<br>p=0.192	100.00%	89.1%<br>p=0.182
	**Others**	54<br>[43 - 64]	57.0%<br>p=0.212	20.9%<br>p=0.045	0.00%	86.5%<br>p=0.926	54<br>[43 - 65]	56.9%<br>p=0.128	19.8%<br>p=0.192	0.00%	86.0%<br>p=0.182
**Steps Variation**	**Overall**	54<br>[43 - 64]	56.90%	20.50%	5.60%	76.10%	54<br>[43 - 64]	56.80%	19.50%	6.20%	76.30%
	**Moderna**	55<br>[44 - 66]	57.3%<br>p=0.682	19.3%<br>p=0.050	4.8%<br>p=0.054	75.6%<br>p=0.441	56<br>[44 - 66]	57.3%<br>p=0.561	18.3%<br>p=0.059	6.0%<br>p=0.699	75.5%<br>p=0.267
	**Pfizer/BioNTech**	53<br>[42 - 63]	56.7%<br>p=0.682	21.4%<br>p=0.050	6.1%<br>p=0.054	76.5%<br>p=0.441	53<br>[42 - 64]	56.5%<br>p=0.561	20.4%<br>p=0.059	6.3%<br>p=0.699	76.8%<br>p=0.267
	**Prev. Positive**	51<br>[40 - 60]	61.4%<br>p=0.112	25.3%<br>p=0.035	100.00%	77.8%<br>p=0.505	52<br>[42 - 61]	62.0%<br>p=0.058	23.1%<br>p=0.105	100.00%	78.7%<br>p=0.309
	**Others**	54<br>[43 - 64]	56.7%<br>p=0.112	20.2%<br>p=0.035	0.00%	76.0%<br>p=0.505	55<br>[43 - 65]	56.5%<br>p=0.058	19.3%<br>p=0.105	0.00%	76.1%<br>p=0.309

Supplementary Table 1. Demographic characteristics (age and gender) and device used by individuals in the 4 groups used in the analysis, depending on vaccine type and if previously tested positive to COVID-19. A chi-squared test has been used to evaluate significant changes in the frequency of observation in each group.

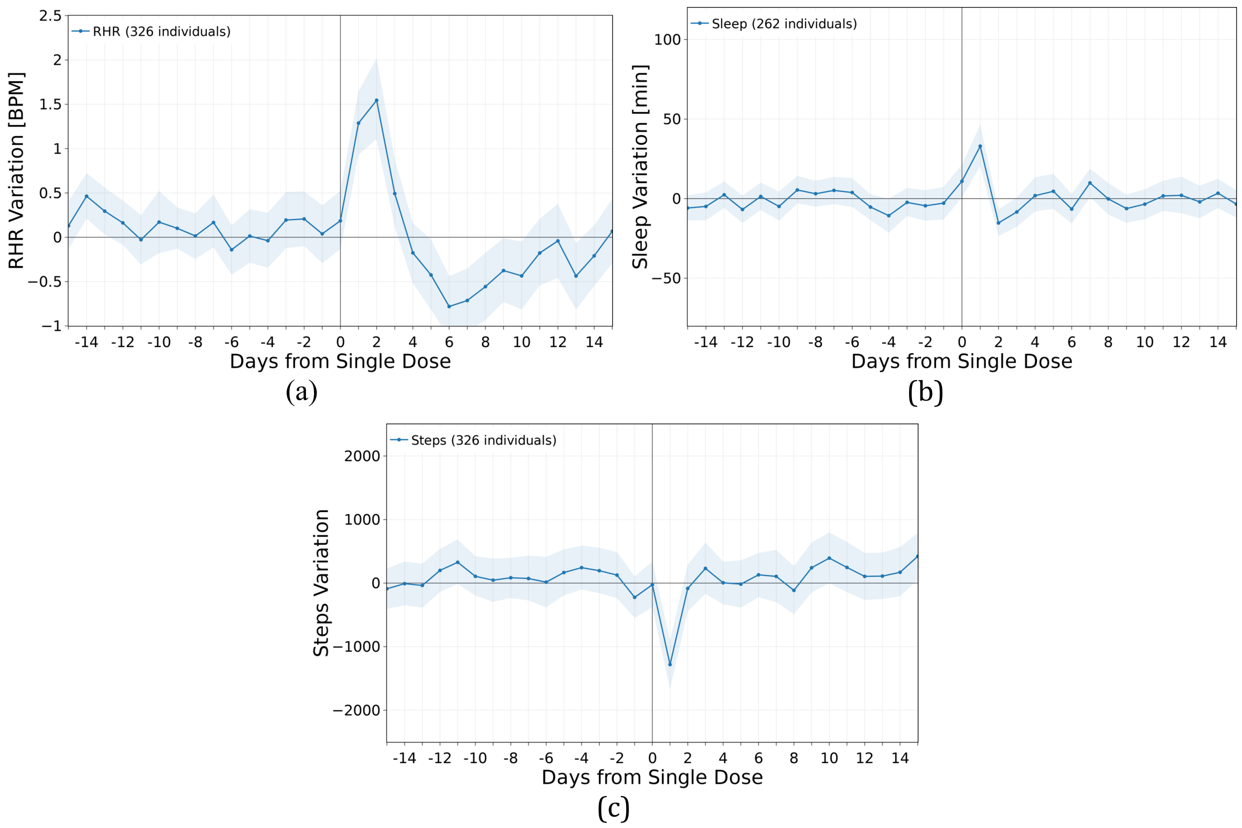

Supplementary Figure 1. Mean and 95% confidence interval of the absolute individual changes in resting heart rate (a), sleep (b) and activity (c) with respect to the individual baseline around the date of vaccination (day 0) for Johnson & Johnson single dose vaccine.

	After First Dose			After Second Dose		
	**Coefficient**	**Std. Error**	**p-value**	**Coefficient**	**Std. Error**	**p-value**
**(Intercept)**	0.251	0.132	0.057	0.757	0.141	< 0.001
**Normalized Age**	0.008	0.126	0.947	-0.411	0.135	0.002
**Gender (1 if Male)**	-0.156	0.066	0.017	0.028	0.070	0.693
**Prev. Covid (1 if Positive)**	0.379	0.141	0.007	-0.193	0.143	0.176
**Vaccine (1 if Moderna)**	0.195	0.065	0.003	0.427	0.069	< 0.001
**Device (1 if Apple)**	0.021	0.075	0.778	0.338	0.080	< 0.001

Supplementary Table 2. Coefficients with 95% confidence interval and associated significance for a multiple regression model to predict the average RHR change after each dose of a mRNA vaccine. Age has been normalized with respect to the population median.
