# Supplementary material for: Inter-individual variation in objective measure of reactogenicity following COVID-19 vaccination via smartwatches and fitness bands
Source: NPJ Digit Med. 2022 Apr 19;5:49. doi: 10.1038/s41746-022-00591-z (PMC9019018; doi:10.1038/s41746-022-00591-z)
Supplement: Supplementary file 2 — Reporting Summary [file 41746_2022_591_MOESM2_ESM.pdf]

nature research

Corresponding author(s): Giorgio Quer

Last updated by author(s): 23/2/22

Reporting Summary

Nature Research wishes to improve the reproducibility of the work that we publish. This form provides structure for consistency and transparency in reporting. For further information on Nature Research policies, see our Editorial Policies and the Editorial Policy Checklist.

Statistics

For all statistical analyses, confirm that the following items are present in the figure legend, table legend, main text, or Methods section.

n/a	Confirmed	
☐	☒	The exact sample size (n) for each experimental group/condition, given as a discrete number and unit of measurement
☐	☒	A statement on whether measurements were taken from distinct samples or whether the same sample was measured repeatedly
☐	☒	The statistical test(s) used AND whether they are one- or two-sided<br>*Only common tests should be described solely by name; describe more complex techniques in the Methods section.*
☐	☒	A description of all covariates tested
☐	☒	A description of any assumptions or corrections, such as tests of normality and adjustment for multiple comparisons
☐	☒	A full description of the statistical parameters including central tendency (e.g. means) or other basic estimates (e.g. regression coefficient) AND variation (e.g. standard deviation) or associated estimates of uncertainty (e.g. confidence intervals)
☐	☒	For null hypothesis testing, the test statistic (e.g. F, t, r) with confidence intervals, effect sizes, degrees of freedom and P value noted<br>*Give P values as exact values whenever suitable.*
☒	☐	For Bayesian analysis, information on the choice of priors and Markov chain Monte Carlo settings
☒	☐	For hierarchical and complex designs, identification of the appropriate level for tests and full reporting of outcomes
☒	☐	Estimates of effect sizes (e.g. Cohen's d, Pearson's r), indicating how they were calculated

Our web collection on statistics for biologists contains articles on many of the points above.

Software and code

Policy information about availability of computer code

Data collection	The MyDataHelps smartphone based app was developed by CareEvolution, and it includes the DETECT study from March 25, 2020 to September 12, 2021. The participants have agreed to share the historical data collected by their eligible devices, including heartrate, sleep and activity measures. On first use, the app records self-reported age, gender and location. With continued use the users are encouraged to share additional information through the completion of targeted surveys, including COVID-19 test outcomes and vaccination information. Data from the participants is automatically uploaded to a protected server.
Data analysis	Analyses were carried out using Python version 3.8.5. The Python packages pandas version 1.1.2 and numpy version 1.19.1 have been used for data processing. Statistical tests and p-values have been evaluated using the Python package scipy version 1.5.2.

For manuscripts utilizing custom algorithms or software that are central to the research but not yet described in published literature, software must be made available to editors and reviewers. We strongly encourage code deposition in a community repository (e.g. GitHub). See the Nature Research guidelines for submitting code & software for further information.

Data

Policy information about availability of data

All manuscripts must include a data availability statement. This statement should provide the following information, where applicable:

- Accession codes, unique identifiers, or web links for publicly available datasets
- A list of figures that have associated raw data
- A description of any restrictions on data availability

All interested investigators will be allowed access to the analysis data set after approval of a proposal by a responsible authority at Scripps and with a data access agreement, pledging to not re-identify individuals or share the data with a third party. All data inquiries should be initially addressed to the corresponding author.

Field-specific reporting

Please select the one below that is the best fit for your research. If you are not sure, read the appropriate sections before making your selection.

☒ Life sciences ☐ Behavioural & social sciences ☐ Ecological, evolutionary & environmental sciences

For a reference copy of the document with all sections, see nature.com/documents/nr-reporting-summary-flat.pdf

Life sciences study design

All studies must disclose on these points even when the disclosure is negative.

Sample size	DETECT is an app-based longitudinal prospective study which has enrolled 39,701 individuals so far from the United States (from March 25, 2020 to September 12, 2021) who have donated their wearable data, self-reported symptoms when ill, viral testing results and vaccination dates/type. Among DETECT participants, 7,298 have reported receiving at least one dose of the vaccine (7,298 received an mRNA vaccine, and 5,674 provided adequate data from the peri-vaccine period for analysis). We applied bootstrap resampling method with 10,000 independent iterations to estimate the uncertainty of the outcomes due to the sample size. Due to the observational nature of the study, we did not perform any statistical analysis to predetermine the sample size, which was enforced by the number of active participants enrolled.
Data exclusions	Among the 7,728 individuals who have reported receiving at least one dose of the vaccine, we excluded 437 individuals who had been vaccinated with the single dose Janssen vaccine since they were too few to allow for a meaningful comparison (their data is reported in the Supplement).<br>We have included in the analysis individuals wearing a Fitbit device (76%) and an Apple watch (20%), while 152 individuals with other devices were not included. We also excluded 75 participants who reported a vaccine date before Dec. 11, 2020, and 16 participants who did not report age or gender. Individuals were excluded if they had less than 4 days of recording in the 2 weeks before dose 1 vaccination, or less than 3 of the 5 days after vaccination, or less than 14 days during the baseline period (from 60 days to 7 days before vaccination). A number of individuals were excluded in the calculation of RHR (1,552), sleep (2,598) and activity (1,535) metrics because of missing data.<br>The data exclusion criteria for the analysis were pre-established. Due to the nature of the study, only individuals owning a smartwatch or activity tracker device were able to be enrolled. No data samples that were eligible for the analysis has been excluded.
Replication	The study has not been replicated, due to the nature of the data collection process, requiring the enrollment of thousands of individuals. The methods have been described in detail in the paper with all the software packages adopted, so it is possible to replicate the study by accessing a similar but independent dataset.
Randomization	Randomization was not relevant for this study, as participants have not been divided in two or more cohorts at the beginning of the study. The study focuses on the discrimination between individuals who tested positive or negative to COVID-19, so no randomization is needed.
Blinding	The group allocation was solely related to the data shared by the participants. Due to the observational nature of the study, the investigators were not involved in any arbitrary group allocation.

Reporting for specific materials, systems and methods

We require information from authors about some types of materials, experimental systems and methods used in many studies. Here, indicate whether each material, system or method listed is relevant to your study. If you are not sure if a list item applies to your research, read the appropriate section before selecting a response.

Materials & experimental systems

n/a	Involved in the study	
☒	☐	Antibodies
☒	☐	Eukaryotic cell lines
☒	☐	Palaeontology and archaeology
☒	☐	Animals and other organisms
☐	☒	Human research participants
☒	☐	Clinical data
☒	☐	Dual use research of concern

Methods

n/a	Involved in the study	
☒	☐	ChIP-seq
☒	☐	Flow cytometry
☒	☐	MRI-based neuroimaging

Human research participants

Policy information about studies involving human research participants

Population characteristics	Between March 25, 2020 and September 12, 2021, our research study enrolled 39,701 individuals with representation from every state in the United States. Among the consented individuals, 7,728 have reported receiving at least one dose of the vaccine (7,298 first dose only, 7,803 both first and second dose, 437 single dose).<br>The most important covariates available at the time of analysis are sex (57% were female), age (median age was 53 (inter quartile range, IQR 42 - 64)), device used by each participants, vaccine type and prior COVID-19 infection (5%).

Recruitment	Any person living in the United States over the age of 18 years old is eligible to participate in the DETECT study by downloading the iOS or Android research app, MyDataHelps. Scripps Research, along with outreach partners, conducted a multi-faceted outreach campaign including social media, educational web-based content, email outreach and in-app notifications to recruit participants for the DETECT Study. The study was also featured in several national media outlets, which increased visibility to larger section of the U.S. population.
Ethics oversight	The protocol for this study was reviewed and approved by the Scripps Office for the Protection of Research Subjects (IRB 20-7531). All individuals participating in the study provided informed consent electronically.

Note that full information on the approval of the study protocol must also be provided in the manuscript.
